# Malignant melanoma in the stomach treated with endoscopic submucosal dissection: a case report

**DOI:** 10.1097/MS9.0000000000000207

**Published:** 2023-02-07

**Authors:** Daisuke Suto, Masashi Yoshida, Takaaki Otake, Yosuke Osawa, Hidehiko Yamada, Kiichi Sato, Takayuki Akita, Hironori Ohdaira, Yutaka Suzuki, Yutaka Kohgo

**Affiliations:** a Department of Gastroenterology; b Department of Surgery, International University of Health and Welfare Hospital, 537-3 Iguchi, Nasushiobara, Tochigi, Japan

**Keywords:** delayed recurrence, endoscopic submucosal dissection, esophagogastroduodenoscopy, malignant melanoma, stomach

## Abstract

The initial appearance of malignant melanoma localized in the stomach has never been reported previously. We encountered a patient with gastric melanoma in the stomach, which was histologically confirmed to be confined to the mucosa.

**Case Presentation:**

The patient, when in her 40s, had undergone surgery for malignant melanoma of the left heel. However, there were no detailed records of pathological findings. The patient had a 4-mm black elevated lesion in her stomach observed on esophagogastroduodenoscopy after the eradication of *Helicobacter pylori.* A year later, esophagogastroduodenoscopy showed that the lesion had increased to 8 mm. A biopsy was performed, but no malignancy was found; the patient continued to be followed up. Esophagogastroduodenoscopy performed at the 2-year follow-up revealed that the melanotic lesion had increased to 15 mm, and biopsy was performed and revealed a malignant melanoma.

**Clinical Discussion:**

Endoscopic submucosal dissection was performed for gastric malignant melanoma. The margin of the resected malignant melanoma was negative; vascular and lymphatic invasions were not observed, and the lesion was confined to the mucosa.

**Conclusion:**

We suggest that even if the first biopsy of a melanotic lesion shows no evidence of malignancy, the lesion should be closely monitored. This is the first reported case of endoscopic submucosal dissection of localized gastric malignant melanoma confined to the mucosa.

Highlights

- Development of malignant melanoma in the stomach was observed for 2 years.
- The article reports the first case of endoscopic submucosal dissection of gastric malignant melanoma.
- Lesions should be closely monitored even if the initial biopsy shows no melanoma.

## Introduction

The initial appearance of malignant melanoma in the stomach has never been previously reported. Malignant melanoma has been found to metastasize to all organs, and is the most common carcinoma metastasizing to the gastrointestinal tract^1^. The median survival of patients with gastrointestinal metastases of malignant melanoma has been reported to be 1 year^2^. Herein, we describe a case of gastric malignant melanoma that was endoscopically followed up for 2 years without any particular symptoms, and was managed successfully with endoscopic submucosal dissection (ESD) after another 1 year. The case has been reported in line with the Surgical CAse REport (SCARE) checklist^3^.

## Presentation of case

**Figure 1:**
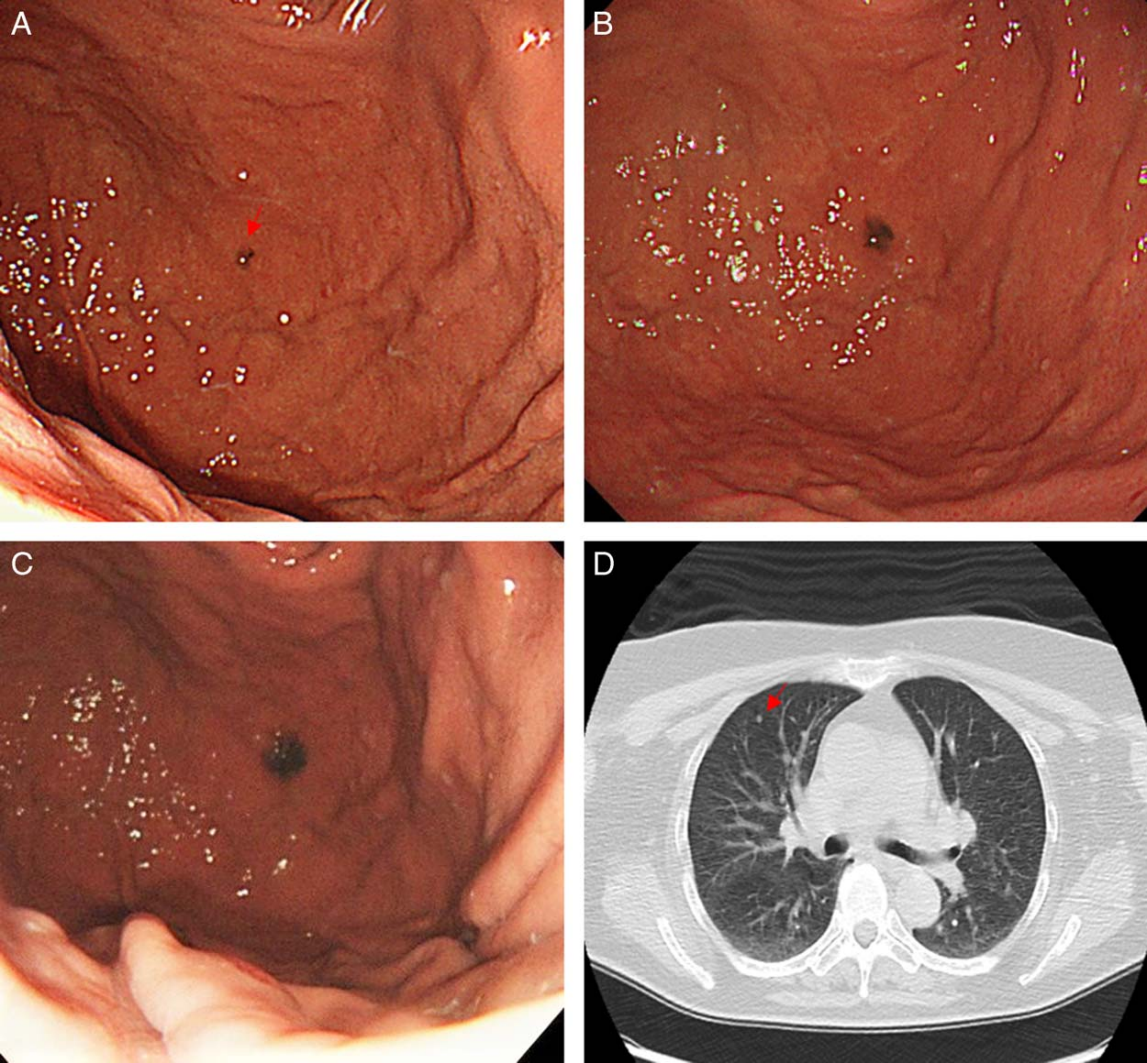
Endoscopic changes over time and computed tomography images. (A) A black elevated lesion 4 mm in size as seen on the anterolateral wall of the fundus. Red arrows indicate lesion. (B) Twelve months after the first esophagogastroduodenoscopy: enlargement of the black elevated lesion to 8 mm. (C) Twenty-four months after the first esophagogastroduodenoscopy: the lesion was further enlarged to 15 mm. (D) Computed tomography scan shows micronodules in the lungs. Red arrows indicate nodules.

**Figure 2:**
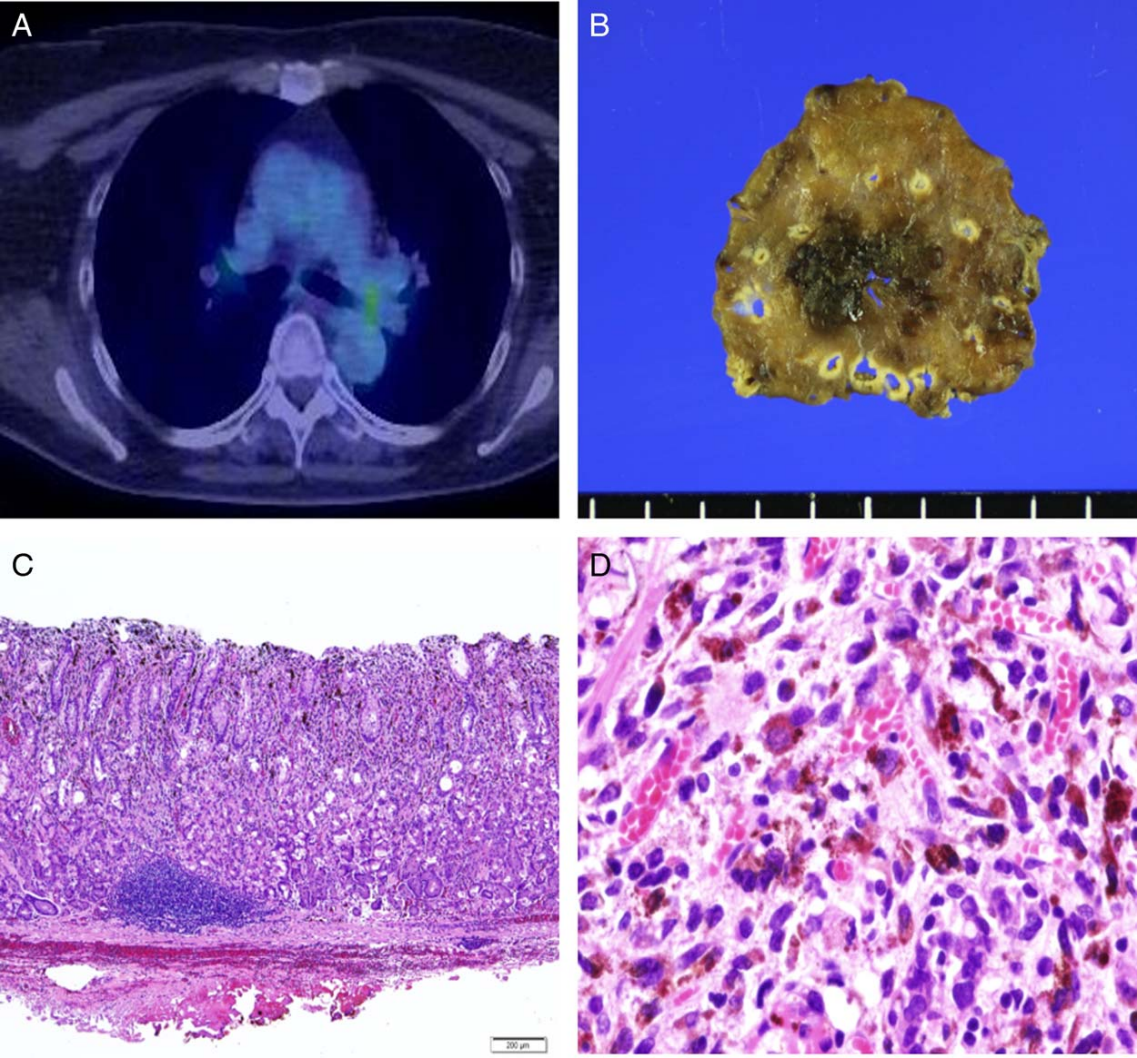
Positron emission tomography–computed tomography images and endoscopic submucosal dissection specimens and hematoxylin and eosin (H&E) stained sections. (A) Positron emission tomography–computed tomography shows no accumulation in the lung nodule shadow noted on computed tomography. (B) Endoscopic resection specimen. (C) Pathological examination of the resection specimen showed no vascular and lymphatic invasion (H&E staining, 4×). (D) The strong enlargement shows tumor cells with vacuolar degeneration and nuclear enlargement (H&E staining, 40×).

**Figure 3:**
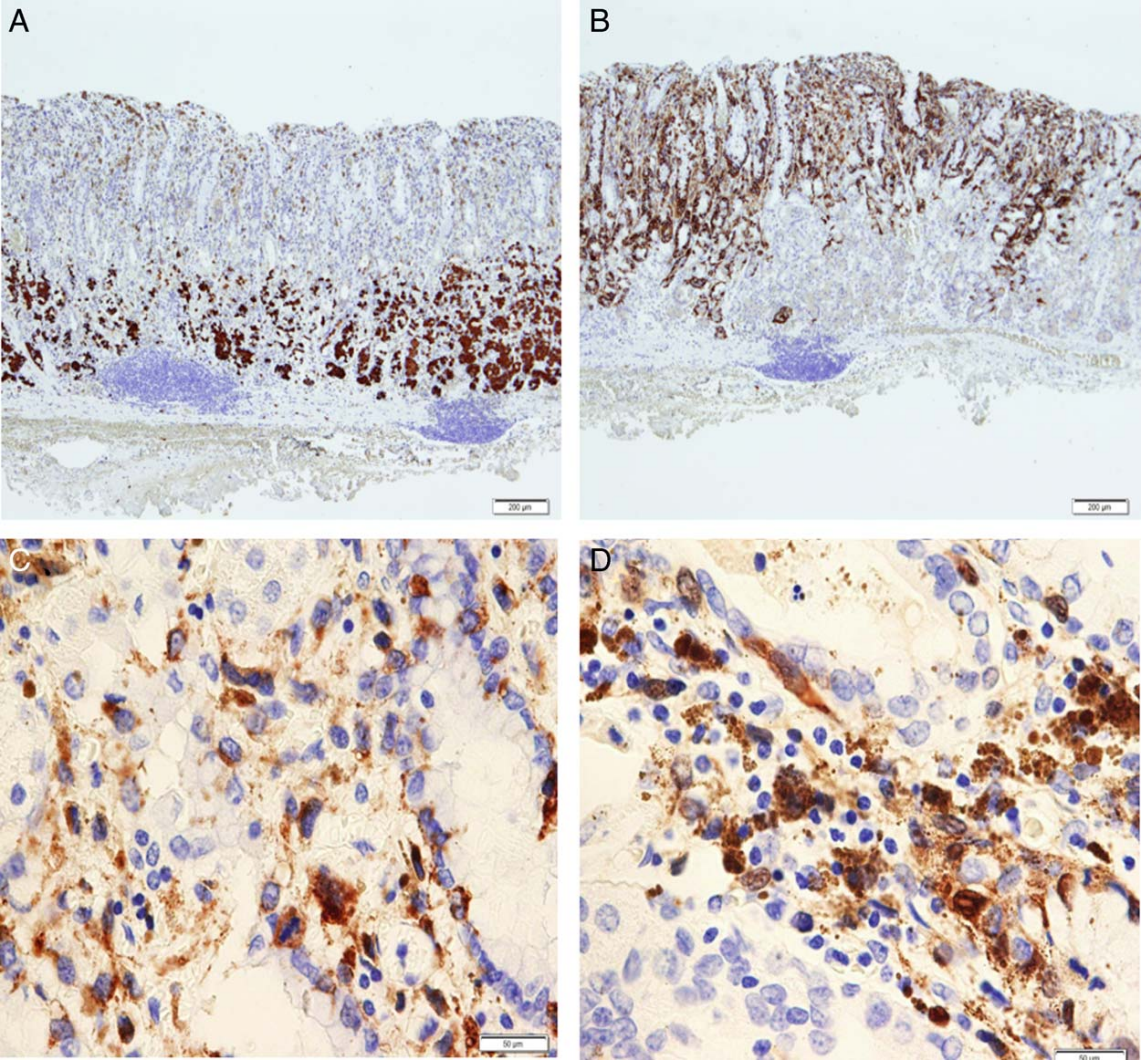
Immunohistochemistry specimen for malignant melanoma. (A) Pathological examination of the specimen showed that the c-kit was localized in the mucosa (scale bar, 200 μm). (B) Pathological examination of the resection specimen showed that melanin A was confined to the mucosa (scale bar, 200 μm). (C) Tumor cells were positive for HMB-45 immunostaining (scale bar, 50 μm). (D) Tumor cells were positive for S-100 immunostaining as well (scale bar, 50 μm).

A woman in her 60s presented to the hospital with a complaint of heartburn after being treated for *Helicobacter pylori* eradication. When in her 40s, she had undergone surgery for malignant melanoma of the left heel. After the malignant melanoma was treated, computed tomography (CT) scans were performed at intervals of 6 months to 1 year for 5 years to monitor the possible appearance of new lesions. However, there were no detailed records of pathological findings. Esophagogastroduodenoscopy showed a black, elevated lesion with a well-defined border of ∼4 mm on the anterior wall of the stomach fundus (Fig. 1A; 2 years before ESD). One year later, esophagogastroduodenoscopy showed that the initial lesion had increased to ∼8 mm with a slightly indistinct border (Fig. 1B; 1 year before ESD). A biopsy was performed; however, no diagnosis of malignant melanoma was made, and the patient was kept under observation. One year later, esophagogastroduodenoscopy revealed that the lesion had enlarged to 15 mm in size (Fig. 1C; the year of ESD). Biopsy revealed malignant melanoma confined to the mucosa; however, laboratory tests showed no abnormalities. CT scan from the chest to the pelvic region did not show tumors, thickening of the gastric wall, or lymph node metastasis, but detected a pulmonary nodule of ∼3 mm in diameter (Fig. 1D). Colonoscopy and brain magnetic resonance imaging showed no abnormality, and positron emission tomography–computed tomography presented no accumulation in the pulmonary nodules detected on CT (Fig. 2A). We consulted a respiratory surgeon about the pulmonary nodule; however, it was difficult to determine whether the lesion was a metastatic tumor because it was only 3 mm in diameter, and the nodule was too small to be resected or biopsied. However, in contrast to the gastric lesions, the nodular lesions in the lung did not expand over the course of the disease. In this case, we used both indigo spraying and narrowband imaging to determine the extent of the malignant melanoma, and it was consistent with the black area noted by ordinary observation. We performed an ESD to remove the malignant melanoma in the stomach (Fig. 2B). We made a mark very close to the tumor, carefully performed precutting around the marking, and successfully obtained a tumor margin of 5 mm. The distribution of the malignant melanoma was consistent with the black elevated lesions of the ESD specimens. The tumor was confined to the epithelium without vascular and lymphatic invasion (Fig. 2C). Hematoxylin and eosin-stained sections showed tumor cells with vacuolar degeneration and nuclear enlargement (Fig. 2D). On immunohistological analysis, the lesion tested positive for c-kit (Fig. 3A), Melan A (Fig. 3B), HMB-45 (Fig. 3C), and S-100 (Fig. 3D). The margin of the resected tissue was negative both horizontally and vertically. Therefore, it was diagnosed as a malignant melanoma localized in the mucosa. Esophagogastroduodenoscopy was performed 2 months, 8 months, and 1 year after the ESD for course observation.

The study was approved by an appropriate ethics committee. Written informed consent was obtained from the patient to publish this case report and any accompanying images.

## Discussion

This is the first reported case of ESD of gastric malignant melanoma confined to the mucosa. A keyword search on PubMed for gastric malignant melanoma and ESD revealed no references. Malignant melanoma of the gastrointestinal tract is known to often metastasize from primary skin tumors: 50% to the small intestine, 30% to the colon, and 20% to the rectum. Metastasis in the stomach is rare^4^. Nevertheless, at least 44 cases of primary malignant melanoma have been reported in the past^5^.

Crowley *et al*.^6^ have reported that among 651 patients who were recurrence-free for 10 years after surgery for primary cutaneous malignant melanoma, 168 (25.8%) experienced subsequent recurrence. In this case, the patient developed a gastric lesion 20 years after the surgical removal of cutaneous melanoma. Even though the possibility of it being a primary melanoma of the stomach cannot be excluded, it is more likely to be a secondary delayed recurrence. Previous reports have stated that gastrointestinal malignant melanomas can be classified as either submucosal tumor-like or primary carcinomatous tumors^7^,^8^. In the present case, the lesion appeared to be a delayed recurrence, but the morphology of the lesion was that of a primary carcinomatous tumor, and it presented as a mildly elevated 0-IIa lesion according to the Japanese classification of gastric carcinoma^9^. Regarding endoscopic resection for gastric malignant melanoma, a case of endoscopic full-thickness resection for gastric metastatic malignant melanoma has been reported previously^10^. However, this is the first case of ESD for malignant melanoma. In our case, biopsy images showed that the melanoma was confined to the mucosa, and the CT scan showed no evidence of gastric wall thickening or lymph node metastasis. The hospital ethics committee discussed the case and recommended ESD for the malignant melanoma according to the usual procedure for early gastric cancer. Pathological findings showed that the malignant melanoma was confined to the mucosa, with negative margins, and negative for vascular and lymphatic invasion; therefore, the resection was locally curative, which does not eradicate the possibility of future recurrence. The patient’s postoperative course after the ESD was stable, and esophagogastroduodenoscopy at 2 months, 8 months, and 1-year follow-up showed no recurrence. Although ESD may be considered a treatment option for malignant melanoma less than 20 mm in size, the postoperative follow-up should be for a longer period. However, no conclusion will be provided until evidence accumulates on similar cases.

Furthermore, in the present case, a c-kit examination was performed to investigate the possibility of metastasis from the left heel; the c-kit was positive in this case. C-kit-positive cases have been reported to be more common in mucosal and acral types, including the left heel^11^. Since this is the first case report of submucosal dissection for malignant melanoma, the positivity of the c-kit cannot be discussed further. The significance of c-kit positivity may become clear as similar cases accumulate in the future.

In this case, we were able to follow the course of malignant melanoma of the stomach for 2 years. Although it was considered a complete resection histologically, whether ESD is effective for malignant melanoma remains unclear. The lung nodules and abdomen will be carefully monitored, and in case of any enlargement of the nodules or the appearance of new lesions, treatment will be indicated. In this patient, a 2-year follow-up enabled us to diagnose malignant melanoma.

Therefore, if a black nodule is observed and biopsy does not indicate malignancy, follow-up is required. In the present case, the melanoma was successfully treated after 2 years of follow-up endoscopy; however, we advise that strict follow-up should be performed if similar cases are encountered in the future.

## Conclusion

In conclusion, we report the first case of ESD for gastric malignant melanoma. The resection was locally curative. Black elevated lesions should be followed up strictly by endoscopy, even if the first biopsy shows no malignant findings.

## Ethical approval

The study was approved by the Ethics Committee of the International University of Health and Welfare Hospital (approval number: 22-B-3).

## Patient consent

Written informed consent was obtained from the patient to publish this case report and any accompanying images.

## Sources of funding

None

## Author contribution

D.S. wrote the manuscript. M.Y., T.O., Y.O., H.Y., K.S., T.A., H.O., Y.S., and Y.K. reviewed the literature and contributed to manuscript drafting. Y.K. was responsible for the revision of the manuscript for important intellectual content. All authors approved the final version of the manuscript to be submitted.

## Conflicts of interest disclosure

The authors declare that they have no financial conflict of interest with regard to the content of this report.

## Research registration unique identifying number (UIN)

None.

## Guarantor

D. Suto, first author; M. Yoshida, senior author.

## Provenance and peer review

Not commissioned, externally peer-reviewed.

## References

[1] Langner C . Secondary tumors of the gastrointestinal tract. Pathologe 2012;33:45–52. 22293789 10.1007/s00292-011-1544-x

[2] Basagoiti ML Vesga F Losada J . Gastric metastasis of melanoma. Rev Esp Enferm Dig 1992;82:419–21. 1493061

[3] Agha RA Franchi T Sohrabi C . SCARE Group. The SCARE 2020 guideline: updating consensus Surgical CAse REport (SCARE) guidelines. Int J Surg 2020;84:226–30. 33181358 10.1016/j.ijsu.2020.10.034

[4] Goral V Ucmak F Yildirim S . Malignant melanoma of the stomach presenting in a woman: a case report. J Med Case Rep 2011;5:94. 21388529 10.1186/1752-1947-5-94 PMC3061929

[5] Mellotte GS Sabu D O’Reilly M . The challenge of primary gastric melanoma: a systematic review. Melanoma Manag 2020;7:MMT51. 10.2217/mmt-2020-0009 PMC7724652 33318781

[6] Crowley NJ Seigler HF . Late recurrence of malignant melanoma. Analysis of 168 patients. Ann Surg 1990;212:173–7. 2375648 10.1097/00000658-199008000-00010 PMC1358053

[7] Oda I Kondo H Yamao T . Metastatic tumors to the stomach: analysis of 54 patients diagnosed at endoscopy and 347 autopsy cases. Endoscopy 2001;33:507–10. 11437044 10.1055/s-2001-14960

[8] Wei SC Su WC Chang MC . Incidence, endoscopic morphology and distribution of metastatic lesions in the gastrointestinal tract. J Gastroenterol Hepatol 2007;22:827–31. 17565636 10.1111/j.1440-1746.2006.04532.x

[9] Japanese Gastric Cancer Association. Japanese classification of gastric carcinoma: 3rd English edition. Gastric Cancer 2011;14:101–12. 21573743 10.1007/s10120-011-0041-5

[10] Kratt T Kueper M Boesmueller H . Endoscopic full-thickness resection of gastric metastasis from malignant melanoma by use of a novel over-the-scope device. Gastrointest Endosc 2016;84:368. 26989035 10.1016/j.gie.2016.03.779

[11] Potti A Moazzam N Langness E . Immunohistochemical determination of HER-2/neu, c-Kit (CD117), and vascular endothelial growth factor (VEGF) overexpression in malignant melanoma. J Cancer Res Clin Oncol 2004;130:80–86. 14634801 10.1007/s00432-003-0509-8 PMC12161809

